# Supplementary material for: Assessment of functional diversities in patients with Asthma, COPD, Asthma-COPD overlap, and Cystic Fibrosis (CF)
Source: PLoS One. 2024 Feb 20;19(2):e0292270. doi: 10.1371/journal.pone.0292270 (PMC10878531; doi:10.1371/journal.pone.0292270)
Supplement: S1 File — (DOCX) [file pone.0292270.s001.docx]

**Supporting information captions**

regarding

Assessment of functional diversities in patients with Asthma, COPD, Asthma-COPD overlap, and Cystic Fibrosis (CF)

Richard Kraemer, Florent Baty, Hans-Jürgen Smith, Stefan Minder, Sabina Gallati,

Martin H. Brutsche, and Heinrich Matthys

**Genotyping**

Genomic DNA was extracted from peripheral blood cells using standard procedures. Then, CF patients were genotyped by a SSCP/HD scanning method followed by direct sequencing of the variants (detection rate 97-98%) as previously described [[45](#_ENREF_45), [69](#_ENREF_69)]. Or all 27 exons (including exon-intron boundaries) as well as the promoter region of the *CFTR* gene (NM_000492.4) were analyzed using Sanger or high-throughput sequencing (Illumina MiSeq, NextSeq). The analysis of the sequencing data was carried out with the software Sequence Pilot (JSI medical systems), Sophia DDM^TM^ (Sophia Genetics SA), and VarSome Clinical (Saphetor). Multiplex Ligation-dependent Probe Amplification (SALSA MLPA probe mix P091, MRC Holland) was used to test for large deletions and duplications.

**Pulmonary function procedures**

The order of maneuvers was as follows: In a first step measurements of airway dynamics giving by the specific aerodynamic airway conductance (sWOB), the effective, specific airway resistance (sR_eff_), and the determination of the specific total resistance (sR_tot_) were conducted. Since the integral method evaluates changes of airway dynamics concomitantly with changes in the end-expiratory lung volume (EELV) at FRC [[32](#_ENREF_32), [34](#_ENREF_34), [50](#_ENREF_50), [70-72](#_ENREF_70)], it was important that parameters of airway dynamics are assessed in this first phase of plethysmographic assessment, and hence not influenced by deep inspiration or forced breathing maneuvers or other efforts [[73-76](#_ENREF_73)]. In a second step, measurements of static lung volumes such as functional residual capacity (FRC_pleth_) obtained by 3 shutter-closure maneuvers were applied, closely linked with a third step of slow maximal expiratory and inspiratory maneuver, giving the measures of residual lung volume (RV), inspiratory capacity (IC), vital capacity (VC) and total lung capacity (TLC). Only in a last forth step forced spirometry was recorded and presented as flow-volume-loop and volume-time-trend, deriving the forced expiratory volume in one second (FEV_1_), the forced vital capacity (FVC) and its ratio to FEV_1_ (FEV_1_/FVC) and the forced expiratory flows between 25% and 75% of exhaled FVC (FEF_25-75_), assessed by standard techniques [[48](#_ENREF_48)]. For the plethysmographic measurements the median of at least 5 single plethysmographic shift volume - tidal flow loops were calculated, and for the indices of the forced breathing parameters the maximum of the 3 valid measurements was taken, as soon as the best and second-best flow-volume loops were comparable in their pattern. Pulmonary function test data of all parameters were assessed in absolute values, as percentage of predicted normal values, and z-transformed accordingly [[52](#_ENREF_52), [53](#_ENREF_53)]. A special export software was developed by PanGas Ltd, Dagmersellen, Switzerland, enabling access to all, routinely stored parameters in every JLab-, Sentry-Suite-databases respectively.

**Assessment of airway dynamics**

The assessment of changes in airway caliber to estimate airway function in human subjects and patients with lung disease is best performed by measuring airway resistance using a whole-body plethysmograph, originally introduced by Dubois et al. [[77](#_ENREF_77), [78](#_ENREF_78)]. This technique was further adapted by Jaeger and Otis to reach body temperature, ambient pressure, and water saturation (BTPS) conditions. Changes in in the caliber of the large, conducting airways are represented by the recording of specific airway loops (sR_aw_-loops), consisting of the shift volume (V_pleth_) and the tidal flow (V’) plot. The lung volume, and hence the resting level of end-expiratory lung volume (EELV) at functional residual capacity (FRC_pleth_) is logically altered in the presence of marked peripheral airway obstruction or pulmonary hyperinflation. It follows that plethysmography offers measurements of airway dynamics in close relationship to FRC_pleth_, and hence the EELV, which has considerable advantages in the detection of functional deficits compared to measurements of simple spirometry, impulse oscillometric (IOS) or interrupter resistance (R_int_) measurements.

Until recently, and still used in many plethysmographs, sR_aw_-loops are approximated routinely by two-point analyses creating a straight line throughout the loop.

$${sR}_{aw}=\frac{{\Delta V}_{pleth}}{V'}\cdot(P_{amb}-P_{H2O})$$

Absolute lung volume FRC_pleth_ is measured by applying a shutter maneuvre and analysing the occlusion-pressure curve.

$${FRC}_{pleth}=\frac{{\Delta V}_{pleth}}{P_{m}}\cdot\left( P_{amb}-P_{H2O} \right)$$

Finally R_aw_ can be calculated from the ration of sR_aw_ and FRC_pleth_.

$$R_{aw}=\frac{{sR}_{aw}}{{FRC}_{pleth}}=\frac{{\Delta V}_{pleth}\cdot P_{m}}{V'\cdot{\Delta V}_{pleth}}\cdot\frac{\left( P_{amb}-P_{H2O} \right)}{\left( P_{amb}-P_{H2O} \right)}=\frac{P_{m}}{V'}$$

The term (P_amb_-P_H2O_) reflects the dry air pressure. It follows that R_aw_ is inevitably linked to the value of FRC_pleth_ as a fixed parameter in the equation, and is computed misleadingly low, if a pulmonary hyperinflation is present, and consistently high, if the subject breathes under his natural EELV. Noteworthy is that FRC_pleth_ implicates a shutter occlusion manoeuvre, which is sometimes not tolerated by young children or elderly patients, as well as by patients with severe lung involvement. Moreover, R_aw_ consistently ignores the loop-shaping of the sR_aw_-loop, especially during quiet breathing, and more importantly, if ventilation inhomogeneities are present.

Although numerous parameters of airway resistances can be calculated from plethysmographic measurements [[79](#_ENREF_79)], the most promising approach was proposed by Matthys H. and Orth U. [[80](#_ENREF_80)], defining the so called “effective specific resistance” (sR_eff_) as a ratio of the area of the plethysmographic shift-volume versus tidal volume ($\oint V_{pleth}dV_{T}$), to the area of the flow/volume loop ($\oint V^{'}\mathrm{dV}_{T}$) throughout the entire respiratory cycle. Noteworthy, the integral $\oint{\Delta V}_{pleth}{dV}_{T}$ multiplied by the dry air pressure (P_amb_-P_H2O_) embodies the specific, aerodynamic work of breathing (sWOB) at rest [[80](#_ENREF_80)]. Only advanced computer technology made it possible to assess the two integrals presented for infants, children ^59,60^, as well as for adults [[80](#_ENREF_80), [81](#_ENREF_81)]. The mathematical background computing sR_eff_, sG_eff_ and sWOB, has been presented previously [[34](#_ENREF_34), [82](#_ENREF_82)], showing the following equations:

$$sR_{eff}= P_{amb}-P_{H_{2}O}* \frac{\oint\Delta V_{pleth}dV_{T}}{\oint V'dV_{T}}= \frac{1}{{sG}_{eff}}$$

where (P_amb_-P_H2O_) is the dry air pressure, the integral $\oint V_{pleth}dV_{T}$ the equivalent to the area enclosed by sWOB and the integral $\oint V^{'}{dV}_{T}$ the equivalent to the area of the flow-volume loop. **Fig S1** synoptically represents a print-screen from a plethysmographic measurement of a healthy infants obtained in an infant whole-body plethysmograph [[83](#_ENREF_83)]. It shows a BTPS-compensated plethysmographic loop, which is the flow dependence (V’) from the shift volume (∆V_pleth_), derived from two further loops. The first loop (Fig S1; A) represents the tidal flow-volume curve, and its area as integral $\oint\Delta V'\mathrm{dV}_{T}$ of the **Fig S1.** Print-screen from a plethysmographic measurement of a healthy infant synoptically representing the interaction of two loops, (A) the tidal flow-volume loop and its area as integral $\oint\Delta V'\mathrm{dV}_{T}$, and (B) the plethysmographic shift volume-tidal volume, incorporating the area $\oint{\Delta V}_{\mathrm{pleth}}\mathrm{dV}_{T}$, and hence the sWOB, from which (C) the BTPS-compensated plethysmographic loop, (shift volume (∆V_pleth_) against tidal flow (V’) can be derived, and sR_eff_, be computed.

The second loop (Fig S1; B) characterizes the tidal volume in relation to the plethysmographic shift volume and incorporates the area $\oint{\Delta V}_{\mathrm{pleth}}\mathrm{dV}_{T}$, and hence the sWOB. The determination of the sWOB is a prerequisite and part of the computation of sR_eff_, sG_eff_, respectively (Fig S1; C), as soon as the subject breathes regularly at resting end-expiratory lung volume (EELV). Importantly however, any extended breathing maneuvers prior to spontaneous resting breathing must be avoided, ensuring airway dynamics are assessed under natural breathing conditions e.g., no panting, no breathing against a shutter, no change in the “volume history” [[84-86](#_ENREF_84)]. Normal spontaneous resting breathing is provided, if the subject breaths naturally and regularly at a constant breathing frequency and constant tidal volume (V_T_) at EELV, and hence with lowest individual sWOB.

The content of the sR_aw_-loop presented in the shift volume (V_pleth_) - tidal flow (V’) - plot (**Fig 1**) seems to be rather complex, especially in patients with COPD. The sR_aw_-loop shows the typical pattern of a golf club in the expiratory limb, which is a sign of airflow limitation in the peripheral airways. The sR_eff_-approximation of the sR_aw_-loop and its reciprocal value, the effective, resistive airway conductance (sG_eff_), were proven to be target parameters reflecting small airways function [[26](#_ENREF_26), [32](#_ENREF_32), [87](#_ENREF_87), [88](#_ENREF_88)].

**Defining predictive equations of airway dynamics**

As an enlargement of a previous paper on normative equations of the so called sR_aw_-loop [[58](#_ENREF_58)] the equations for the inspiratory (sWOB_in_, sR_eff_^IN^) and expiratory parts (sWOB_ex_, sR_eff_^EX^) of the sRaw-loop were obtained by a multi-level modelling approach revealed following normative equations:

**sWOB** = EXP(.063 + .117*ln(age) + 1.230*ln(V_T_) - .562*T_I_/T_tot_)
± .082964 (SEE)
(*F*-value: 690, *p*<0.001) (1)

**sWOB_in_** = EXP(-.675 + 0.024*ln(age) + 0.966*ln(V_T_))
± .038671 (SEE)
(*F*-value: 2086, *p*<0.001) (2)

**sWOB_ex_** = EXP(-.636 + 0.063*ln(age) + 0.730*ln(V_T_) – 0.349*T_I_/T_tot_)
± .034237 (SEE)
(*F*-value: 1401, *p*<0.001) (3)

**sR_ef_**_f_ = EXP(1.304 +.084*gender + 0.002*age + 2.001E-5*(height)^2^ – 0.441*ln(V_T_)) ± .085933 (SEE)

*gender: male=0; female=1; (*F*-value: 27.4, *p*<0.001) (4)

**sR_eff_^IN^** = EXP(-1.014 + .008*gender + .344*V_T_/T_I_ - .282*ln(V_T_/T_I_ -.052*ln(V_T_/FRC)) ± .012675 (SEE)
(*F*-value: 20, *p*<0.001) (5)

**sR_eff_^EX^** = EXP(-.634 + .030*gender + .059*ln(age) - .298*ln(V_T_)
± .069861 (SEE)
(*F*-value: 15, *p*<0.001) (6)

**sR_tot_** = EXP(-.387 - .135*ln(V_T_)
± .09591 (SEE)
(*F*-value: 15, *p*<0.001) (7)

**P_0.1_** = EXP (-2.283 + 0.325*ln(age) – 3.536E-5*(age)^2^)
± .06488 (SEE)
(*F*-value: 760, *p*<0.001) (8)

**V_T_/T_I_** = 0.148 – 0.025*gender* – 0.004*age + 0.202*ln(age)
± .0444258 (SEE)
*gender: male=0; female=1; (*F*-value: 131, *p*<0.001) (9)

**T_I_/T_tot_** = 0.431 – 0.011*gender* - 8.058E-6*(age)^2^
+ 3.641E-5*(age)^2^ ± .018294 (SEE)
*gender: male=0; female=1; (*F*-value: 61, *p*<0.001) (10)

**Z_in_^pleth^** = 3.61 + 0.015*gender* + 0.066*ln(age) – 0.736*ln(height) +
1.268E-5*(height)^2^ ± .03086 (SEE)
*gender: male=0; female=1; (*F*-value: 73, *p*<0.001) (11)

**Lung function comparison between the centers**
(Asthma, ACO, COPD)

Relatively small number of subjects per center within the sub-groups of COPD could be a limiting factor regarding the power of potentially discriminating parameters. However, there were no differences when the centers were compared to one another, as presented by **S1 Table.**

**Figure**

**Fig S1.** Print-screen from a plethysmographic measurement of a healthy infant synoptically representing the interaction of two loops, (A) the tidal flow-volume loop and its area as integral $\oint\Delta V'\mathrm{dV}_{T}$, and (B) the plethysmographic shift volume-tidal volume, incorporating the area $\oint{\Delta V}_{\mathrm{pleth}}\mathrm{dV}_{T}$, and hence the sWOB, from which (C) the BTPS-compensated plethysmographic loop, (shift volume (∆V_pleth_) against tidal flow (V’) can be derived, and sR_eff_, be computed.

**Figure**

**Fig S1**


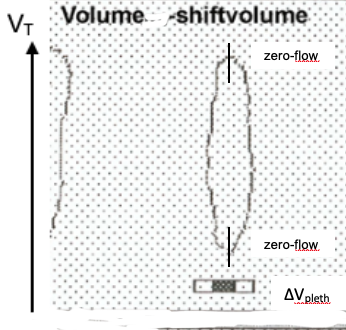

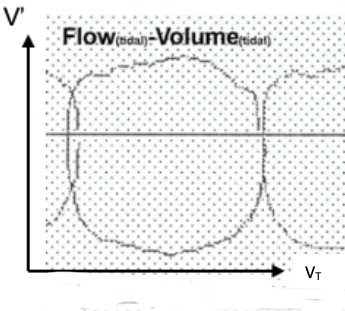


**The work of breathing loop**

$$sWOB=\oint V_{pleth}{dV}_{T}$$

**The breathing cycle**

Tidal flow-tidal volume loop

$$\oint\Delta V'{dV}_{T}$$

**The plethysmographic**

**tidal flow – shift volume loop**


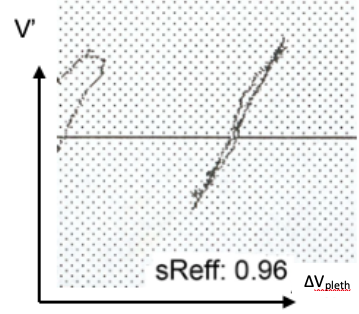


A

B

C

zero-flow

zero-flow

∆V_pleth_

**Volume - shift volume**

**Flow_(tidal)_ – Volume_(tidal)_**

sR_eff_: 0.96 kPa*s

∆V_pleth_

**S1 Table. Values of parameters separated by diagnosis and center of origin**


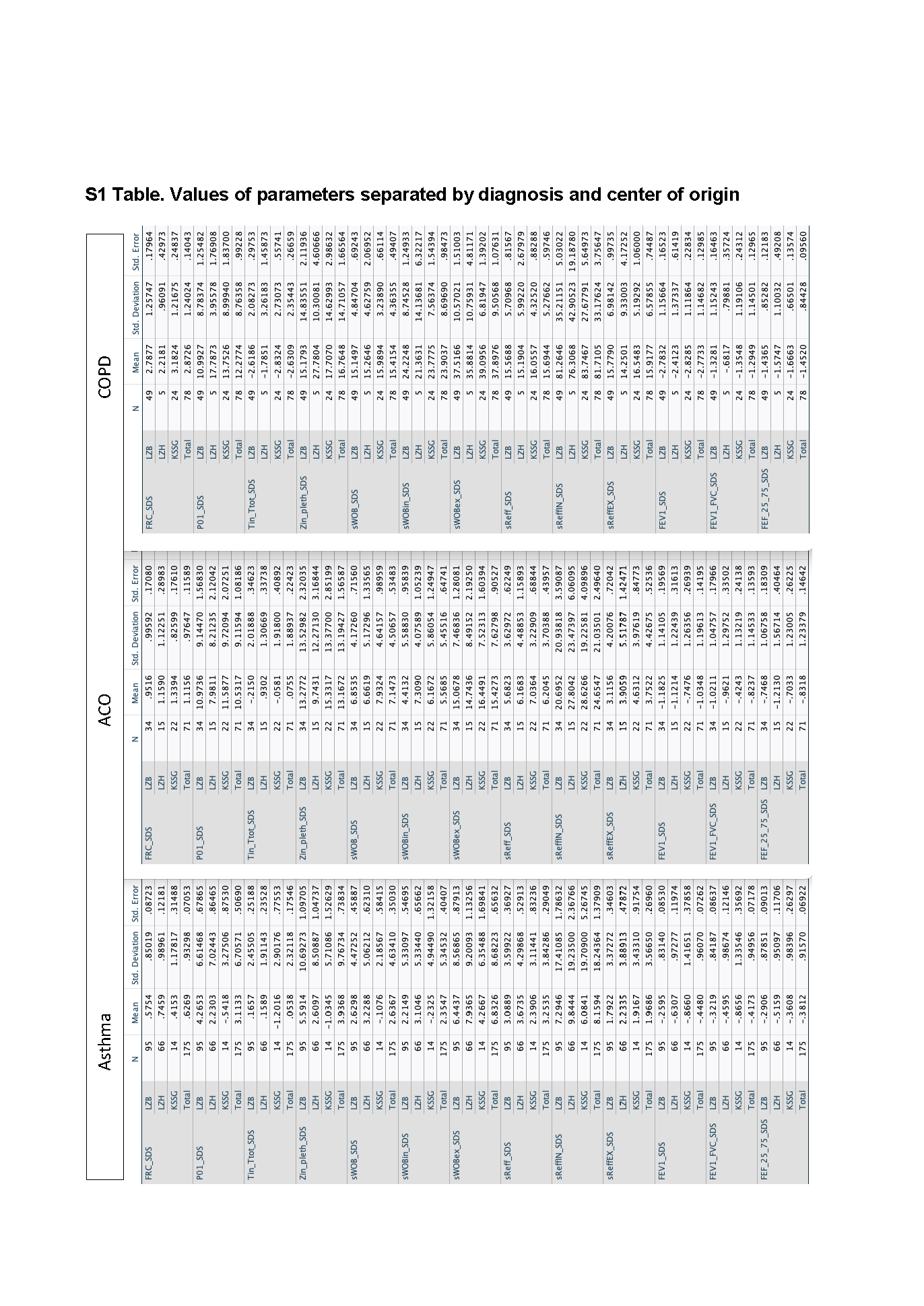


**S2 Table. Minimal Data Set**

**
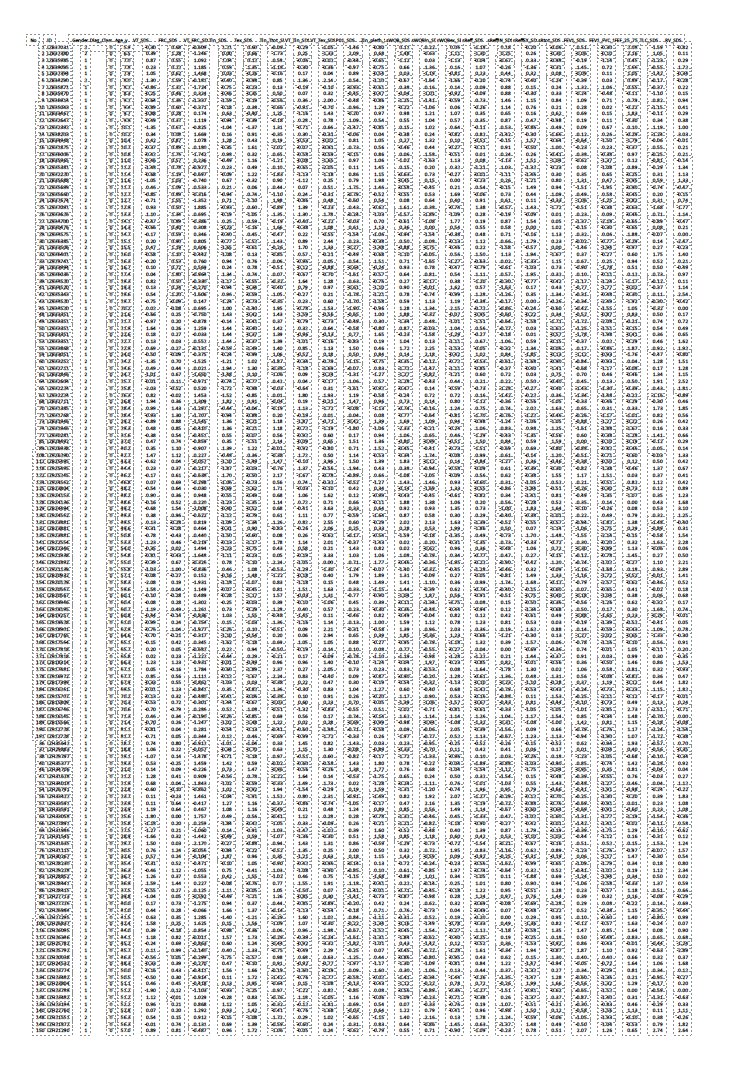
**

**
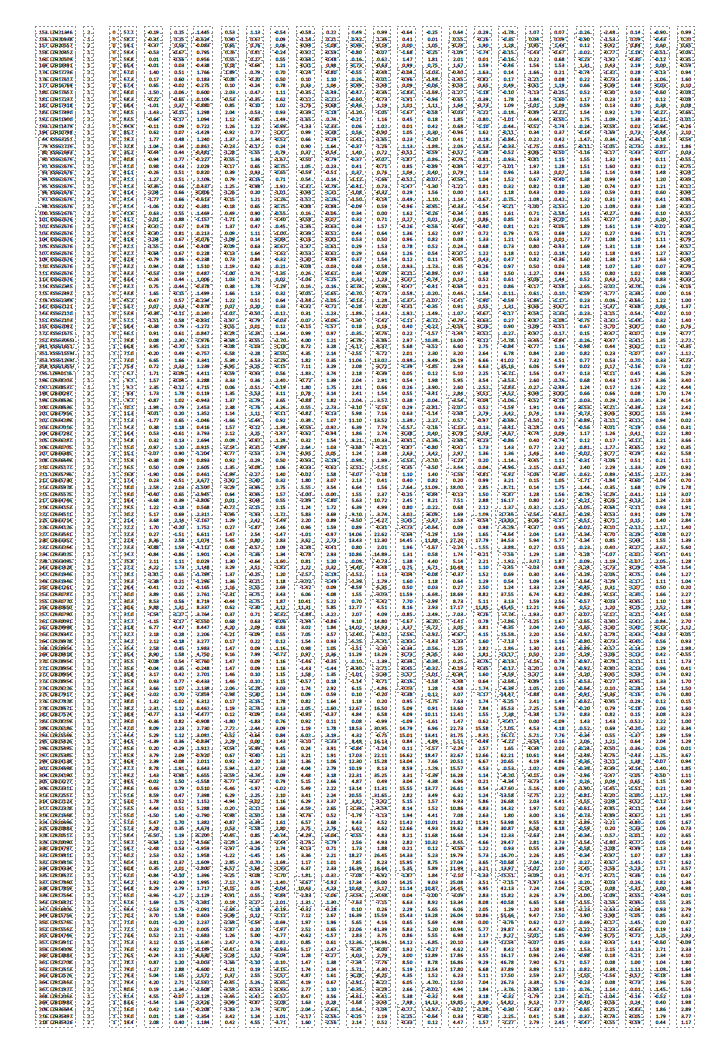
**

**
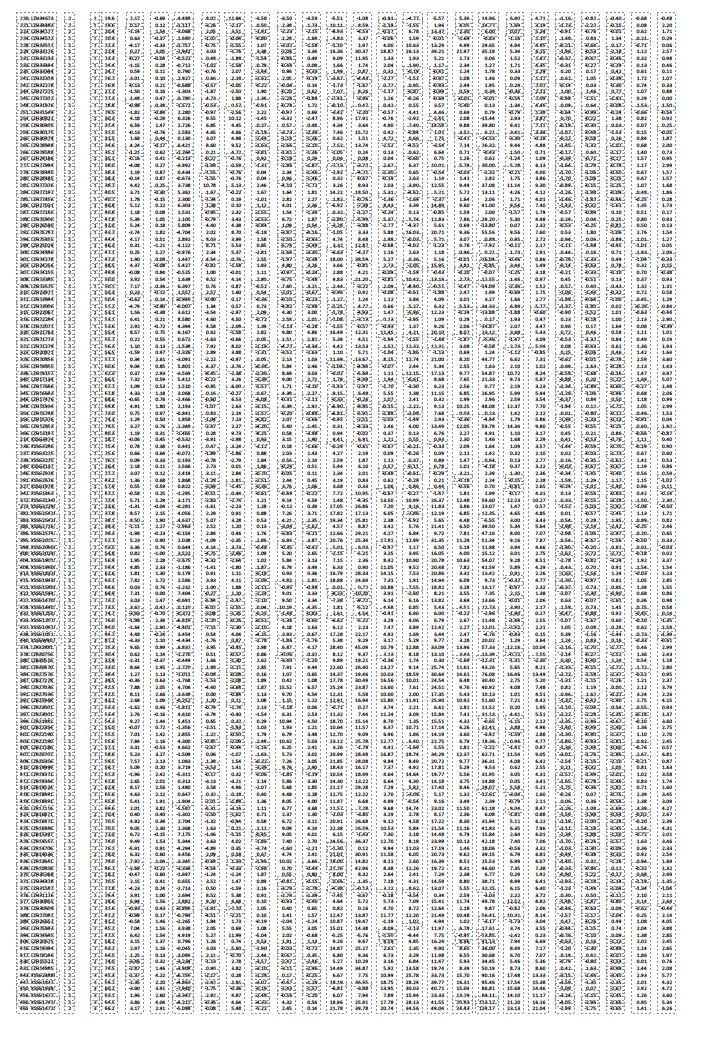
**

**
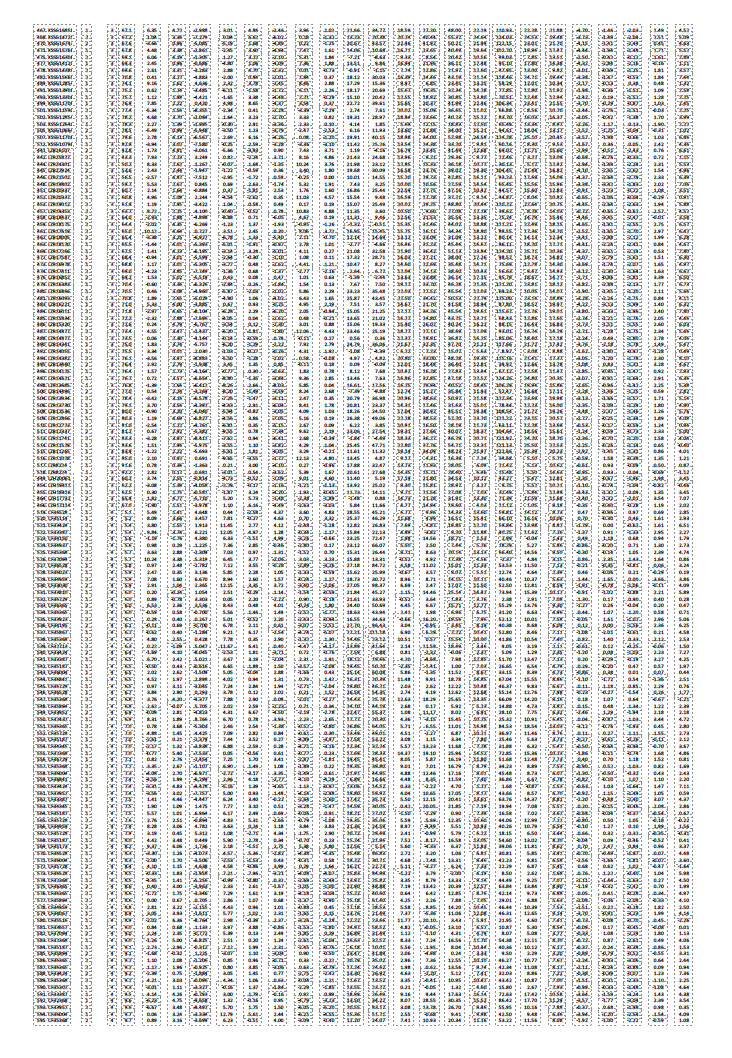
**

**
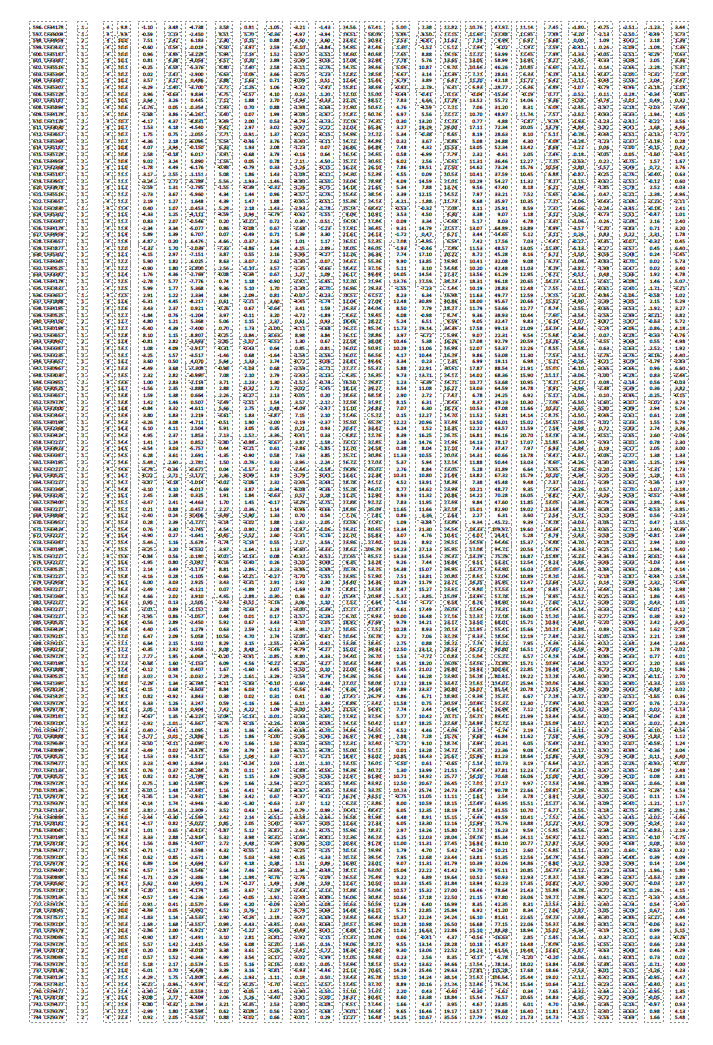
**

**
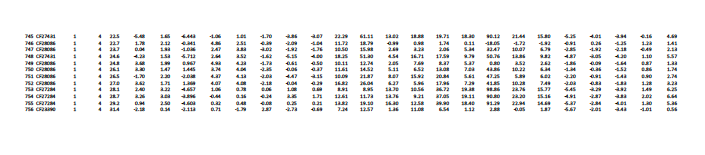
**

**References**

26. Topalovic M, Derom E, Osadnik CR, Troosters T, Decramer M, Janssens W, et al. Airways resistance and specific conductance for the diagnosis of obstructive airways diseases. Respir Res. 2015;16:88, 1-8. Epub 2015/07/22. doi: 10.1186/s12931-015-0252-0. PubMed PMID: 26194099; PubMed Central PMCID: PMCPMC4509748.

32. Kraemer R, Smith HJ, Gardin F, Barandun J, Minder S, Kern L, et al. Bronchodilator Response in Patients with COPD, Asthma-COPD-Overlap (ACO) and Asthma, Evaluated by Plethysmographic and Spirometric z-Score Target Parameters. Int J Chron Obstruct Pulmon Dis. 2021;16:2487-500. Epub 20210901. doi: 10.2147/COPD.S319220. PubMed PMID: 34511893; PubMed Central PMCID: PMCPMC8420556.

34. Kraemer R, Blum A, Schibler A, Ammann RA, Gallati S. Ventilation inhomogeneities in relation to standard lung function in patients with cystic fibrosis. Am J Respir Crit Care Med. 2005;171(4):371-8. Epub 20041105. doi: 10.1164/rccm.200407-948OC. PubMed PMID: 15531750.

45. Liechti-Gallati S, Schneider V, Neeser D, Kraemer R. Two buffer PAGE system-based SSCP/HD analysis: a general protocol for rapid and sensitive mutation screening in cystic fibrosis and any other human genetic disease. Eur J Hum Genet. 1999;7(5):590-8. PubMed PMID: 10439967.

48. Pellegrino R, Viegi G, Brusasco V, Crapo RO, Burgos F, Casaburi R, et al. Interpretative strategies for lung function tests. Eur Respir J. 2005;26(5):948-68. Epub 2005/11/03. doi: 10.1183/09031936.05.00035205. PubMed PMID: 16264058.

50. Goldman M, Smith HJ, Ulmer WT. Lung function testing: Whole-body plethysmography. Eur Respir Mon. 2005;31:15-43.

52. Stocks J, Quanjer PH. Reference values for residual volume, functional residual capacity and total lung capacity. ATS Workshop on Lung Volume Measurements. Official Statement of The European Respiratory Society. Eur Respir J. 1995;8(3):492-506. PubMed PMID: 7789503.

53. Hankinson JL, Odencrantz JR, Fedan KB. Spirometric reference values from a sample of the general U.S. population. Am J Respir Crit Care Med. 1999;159(1):179-87. doi: 10.1164/ajrccm.159.1.9712108. PubMed PMID: 9872837.

58. Kraemer R, Smith HJ, Matthys H. Normative reference equations of airway dynamics assessed by whole-body plethysmography during spontaneous breathing evaluated in infants, children, and adults. Physiol Rep. 2021;9(17):1-13. doi: 10.14814/phy2.15027. PubMed PMID: 34514738; PubMed Central PMCID: PMCPMC8436055.

69. Steiner B, Truninger K, Sanz J, Schaller A, Gallati S. The role of common single-nucleotide polymorphisms on exon 9 and exon 12 skipping in nonmutated CFTR alleles. Hum Mutat. 2004;24(2):120-9. PubMed PMID: 15241793.

70. Matthys H. The assessment of airways obstruction. Bull Physiopathol Respir (Nancy). 1973;9(4):961-78. PubMed PMID: 4770760.

71. Kirkby J, Stanojevic S, Welsh L, Lum S, Badier M, Beardsmore C, et al. Reference equations for specific airway resistance in children: the Asthma UK initiative. Eur Respir J. 2010;36(3):622-9. Epub 2010/02/13. doi: 09031936.00135909 [pii] 10.1183/09031936.00135909. PubMed PMID: 20150205.

72. Kraemer R, Smith HJ, Sigrist T, Giger G, Keller R, Frey M. Diagnostic accuracy of methacholine challenge tests assessing airway hyperreactivity in asthmatic patients - a multifunctional approach. Respir Res. 2016;17(1):154. Epub 2016/11/20. doi: 10.1186/s12931-016-0470-0. PubMed PMID: 27855687; PubMed Central PMCID: PMCPMC5114725.

73. Kapsali T, Permutt S, Laube B, Scichilone N, Togias A. Potent bronchoprotective effect of deep inspiration and its absence in asthma. J Appl Physiol (1985). 2000;89(2):711-20. PubMed PMID: 10926658.

74. Salome CM, Thorpe CW, Diba C, Brown NJ, Berend N, King GG. Airway re-narrowing following deep inspiration in asthmatic and nonasthmatic subjects. Eur Respir J. 2003;22(1):62-8. PubMed PMID: 12882452.

75. Slats AM, Janssen K, van Schadewijk A, van der Plas DT, Schot R, van den Aardweg JG, et al. Bronchial inflammation and airway responses to deep inspiration in asthma and chronic obstructive pulmonary disease. Am J Respir Crit Care Med. 2007;176(2):121-8. doi: 10.1164/rccm.200612-1814OC. PubMed PMID: 17379851.

76. Nensa F, Kotschy-Lang N, Smith HJ, Marek W, Merget R. Assessment of airway hyperresponsiveness: comparison of spirometry and body plethysmography. Adv Exp Med Biol. 2013;755:1-9. Epub 2012/07/25. doi: 10.1007/978-94-007-4546-9_1. PubMed PMID: 22826043.

77. Dubois AB, Botelho SY, Bedell GN, Marshall R, Comroe JH, Jr. A rapid plethysmographic method for measuring thoracic gas volume: a comparison with a nitrogen washout method for measuring functional residual capacity in normal subjects. J Clin Invest. 1956;35(3):322-6. PubMed PMID: 13295396.

78. Dubois AB, Botelho SY, Comroe JH, Jr. A new method for measuring airway resistance in man using a body plethysmograph: values in normal subjects and in patients with respiratory disease. J Clin Invest. 1956;35(3):327-35. PubMed PMID: 13295397.

79. Ulmer WT, Marek W, Rasche K. [Airway resistance curves in obstructive respiratory tract diseases. 8 types of airway resistance curves in spontaneous respiration]. Fortschr Med. 1988;106(33):663-4, 5-6, 7. Epub 1988/11/20. PubMed PMID: 3061907.

80. Matthys H, Orth U. Comparative measurements of airway resistance. Respiration. 1975;32(2):121-34. PubMed PMID: 1118676.

81. Zaiss AW, Matthys H. A multiuser system for whole body plethysmographic measurements and interpretation. Lung. 1990;168 Suppl:1185-92. PubMed PMID: 2117120.

82. Kraemer R, Delosea N, Ballinari P, Gallati S, Crameri R. Effect of allergic bronchopulmonary aspergillosis on lung function in children with cystic fibrosis. Am J Respir Crit Care Med. 2006;174(11):1211-20. Epub 20060907. doi: 10.1164/rccm.200603-423OC. PubMed PMID: 16959918.

83. Kraemer R. Whole-body plethysmography in the clinical assessment of infants with bronchopulmonary diseases. Respiration. 1993;60(1):1-8. PubMed PMID: 8469815.

84. Agostoni E, Mead, J. Statics of the respiratory system. In: Fenn WO, Rahn, H. , editor. Handbook of Physiology. American Physiological Society, Washington D.C. ed1964. p. 411-27.

85. Mead J, Turner JM, Macklem PT, Little JB. Significance of the relationship between lung recoil and maximum expiratory flow. J Appl Physiol. 1967;22(1):95-108. PubMed PMID: 6017658.

86. Duggan CJ, Castle WD, Berend N. Effects of continuous positive airway pressure breathing on lung volume and distensibility. J Appl Physiol (1985). 1990;68(3):1121-6. PubMed PMID: 2187850.

87. Kaminsky DA. What does airway resistance tell us about lung function? Respir Care. 2012;57(1):85-96; discussion -9. doi: 10.4187/respcare.01411. PubMed PMID: 22222128.

88. Santus P, Radovanovic D, Henchi S, Di Marco F, Centanni S, D'Angelo E, et al. Assessment of acute bronchodilator effects from specific airway resistance changes in stable COPD patients. Respir Physiol Neurobiol. 2014;197:36-45. doi: 10.1016/j.resp.2014.03.012. PubMed PMID: 24726342.
