# Supplementary material for: Assessment of functional diversities in patients with Asthma, COPD, Asthma-COPD overlap, and Cystic Fibrosis (CF)
Source: PLoS One. 2024 Feb 20;19(2):e0292270. doi: 10.1371/journal.pone.0292270 (PMC10878531; doi:10.1371/journal.pone.0292270)
Supplement: Attachment — Submitted filename: Response_Letter.docx [file pone.0292270.s002.docx]

PONE-D-23-12620

Assessment of functional diversities in patients with Asthma, Asthma-COPD overlap, and Cystic Fibrosis (CF)

PLOS ONE

Dear Editor,

Dear Dr. Ruud AW Veldhuizen

many thanks for your letter of August 11, announcing that a revision of our paper could improve the chance, that it could be of merit to be accepted and published in PLOS ONE.

We very much appreciate this possibility and are happy to respond to yours and the two Reviewer’s comments.

They have been very helpful for a rigorous revision of our paper.

As requested, please find attached:

- A rebuttal letter that responds to each point raised by the academic editor and reviewer(s) attached at this letter.
- A minimal data set, attached and up-loaded.

The **Figures** have been revised and up-loaded to the Preflight Analysis and Conversion Engine (PACE) digital diagnostic tool

Moreover:

- This letter has been up-loaded as a separate file labeled 'Response to Reviewers'.
- Moreover a marked-up copy of your manuscript that highlights changes made to the original version has been up-loaded, together with
- An unmarked version of your revised paper without tracked changes.

With man thanks and kind regards

R. Kraemer, MD

---

--------

Richard Kraemer, MD

Professor of Paediatrics and Paediatric Pulmonology e.m.

retired Chairman of the Department of Paediatrics, University of Berne

J.V. Widmannstrasse 25

CH-3074 Muri near Berne, Switzerland

+41 79 300 26  53

**From:** [em.pone.0.854605.0459e935@editorialmanager.com](mailto:em.pone.0.854605.0459e935@editorialmanager.com) <[em.pone.0.854605.0459e935@editorialmanager.com](mailto:em.pone.0.854605.0459e935@editorialmanager.com)> on behalf of PLOS ONE <[em@editorialmanager.com](mailto:em@editorialmanager.com)>
**Date:** Friday, 11 August 2023 at 01:43
**To:** Richard Kraemer <[richard.kraemer@quickline.ch](mailto:richard.kraemer@quickline.ch)>
**Subject:** PLOS ONE Decision: Revision required [PONE-D-23-12620] - [EMID:58803a674bea1ce0]

PONE-D-23-12620

Assessment of functional diversities in patients with Asthma, Asthma-COPD overlap, and Cystic Fibrosis (CF)

PLOS ONE

Dear Dr. Kraemer,

Thank you for submitting your manuscript to PLOS ONE. After careful consideration, we feel that it has merit but does not fully meet PLOS ONE’s publication criteria as it currently stands. Therefore, we invite you to submit a revised version of the manuscript that addresses the points raised during the review process.

Please submit your revised manuscript by Sep 24 2023 11:59PM. If you will need more time than this to complete your revisions, please reply to this message or contact the journal office at [plosone@plos.org](mailto:plosone@plos.org). When you're ready to submit your revision, log on to <https://www.editorialmanager.com/pone/> and select the 'Submissions Needing Revision' folder to locate your manuscript file.

Please include the following items when submitting your revised manuscript:

- A rebuttal letter that responds to each point raised by the academic editor and reviewer(s). You should upload this letter as a separate file labeled 'Response to Reviewers'.
- A marked-up copy of your manuscript that highlights changes made to the original version. You should upload this as a separate file labeled 'Revised Manuscript with Track Changes'.
- An unmarked version of your revised paper without tracked changes. You should upload this as a separate file labeled 'Manuscript'.

If you would like to make changes to your financial disclosure, please include your updated statement in your cover letter. Guidelines for resubmitting your figure files are available below the reviewer comments at the end of this letter.

If applicable, we recommend that you deposit your laboratory protocols in protocols.io to enhance the reproducibility of your results. Protocols.io assigns your protocol its own identifier (DOI) so that it can be cited independently in the future. For instructions see: <https://journals.plos.org/plosone/s/submission-guidelines#loc-laboratory-protocols>. Additionally, PLOS ONE offers an option for publishing peer-reviewed Lab Protocol articles, which describe protocols hosted on protocols.io. Read more information on sharing protocols at <https://plos.org/protocols?utm_medium=editorial-email&utm_source=authorletters&utm_campaign=protocols>.

We look forward to receiving your revised manuscript.

Kind regards,

Ruud AW Veldhuizen
Academic Editor
PLOS ONE

**Ademic Editor and Reviewer(s)**

Journal Requirements:

When submitting your revision, we need you to address these additional requirements.

1. Please ensure that your manuscript meets PLOS ONE's style requirements, including those for file naming. The PLOS ONE style templates can be found at

<https://journals.plos.org/plosone/s/file?id=wjVg/PLOSOne_formatting_sample_main_body.pdf> and 
<https://journals.plos.org/plosone/s/file?id=ba62/PLOSOne_formatting_sample_title_authors_affiliations.pdf>

RK.: The paper is now strictly set-up according to the instructions to the authors

2. We noticed you have some minor occurrence of overlapping text with the following previous publication(s), which needs to be addressed:

- <https://www.dovepress.com/front_end/bronchodilator-response-in-patients-with-copd-asthma-copd-overlap-aco--peer-reviewed-fulltext-article-COPD>
- <https://onlinelibrary.wiley.com/doi/10.1111/apm.12915>

In your revision ensure you cite all your sources (including your own works), and quote or rephrase any duplicated text outside the methods section. Further consideration is dependent on these concerns being addressed.

RK.: All overlapping text segments are now cited and referenced

3. In your Data Availability statement, you have not specified where the minimal data set underlying the results described in your manuscript can be found. PLOS defines a study's minimal data set as the underlying data used to reach the conclusions drawn in the manuscript and any additional data required to replicate the reported study findings in their entirety. All PLOS journals require that the minimal data set be made fully available. For more information about our data policy, please see <http://journals.plos.org/plosone/s/data-availability>.

RK.: A completely anonymized “minimal underlying data set” was performed and stored in the Clinical Trial Unit (CTU), Hirslanden, Corporate Office, CH-8152 Glattpark, Switzerland, as well as in the cover letter

Upon re-submitting your revised manuscript, please upload your study’s minimal underlying data set as either Supporting Information files or to a stable, public repository and include the relevant URLs, DOIs, or accession numbers within your revised cover letter. For a list of acceptable repositories, please see <http://journals.plos.org/plosone/s/data-availability#loc-recommended-repositories>. Any potentially identifying patient information must be fully anonymized.

Important: If there are ethical or legal restrictions to sharing your data publicly, please explain these restrictions in detail. Please see our guidelines for more information on what we consider unacceptable restrictions to publicly sharing data: <http://journals.plos.org/plosone/s/data-availability#loc-unacceptable-data-access-restrictions>. Note that it is not acceptable for the authors to be the sole named individuals responsible for ensuring data access.

We will update your Data Availability statement to reflect the information you provide in your cover letter.

4. Please amend either the title on the online submission form (via Edit Submission) or the title in the manuscript so that they are identical.

RK.: Both Titles are the same: “Assessment of functional diversities in patients with Asthma, COPD, Asthma-COPD overlap, and Cystic Fibrosis (CF)”

5. We note that you have included the phrase “data not shown” in your manuscript. Unfortunately, this does not meet our data sharing requirements. PLOS does not permit references to inaccessible data. We require that authors provide all relevant data within the paper, Supporting Information files, or in an acceptable, public repository. Please add a citation to support this phrase or upload the data that corresponds with these findings to a stable repository (such as Figshare or Dryad) and provide and URLs, DOIs, or accession numbers that may be used to access these data. Or, if the data are not a core part of the research being presented in your study, we ask that you remove the phrase that refers to these data.

RK.: The comparison of the data obtained from the 4 center is now given in the “Supporting information captions”

6. Please remove your figure 1 from within your manuscript file, leaving only the individual TIFF/EPS image files, uploaded separately. These will be automatically included in the reviewers’ PDF.

RK.: I cannot follow your intention: Why only remove Figure 1? All 3 Figures have been resized and uploaded to PACE

7. Please upload a new copy of Figure 3 as the detail is not clear. Please follow the link for more information:
<https://blogs.plos.org/plos/2019/06/looking-good-tips-for-creating-your-plos-figures-graphics/>

RK.: Figure 3 was rebuilt and expressed as .tiff.

8. Please include captions for your Supporting Information files at the end of your manuscript, and update any in-text citations to match accordingly. Please see our Supporting Information guidelines for more information: <http://journals.plos.org/plosone/s/supporting-information>.

**Additional Editor Comments:**

As you can note from the reviewer's comments, there is a substantial discrepancy between the assessments of the two reviewers. In revising your manuscript please pay particular attention to the discussion of the manuscript and how the results are interpreted. In addition, please make sure clear definitions of the assessed groups are provided and discussed.

On a separate note, I like to apologize for the long time that has elapsed between your submission and providing you with these reviews.   

**Reviewers' comments:**

Reviewer's Responses to Questions

Comments to the Author

1. Is the manuscript technically sound, and do the data support the conclusions?

The manuscript must describe a technically sound piece of scientific research with data that supports the conclusions. Experiments must have been conducted rigorously, with appropriate controls, replication, and sample sizes. The conclusions must be drawn appropriately based on the data presented.

Reviewer #1: Yes

Reviewer #2: No

2. Has the statistical analysis been performed appropriately and rigorously?

Reviewer #1: Yes

Reviewer #2: Yes

3. Have the authors made all data underlying the findings in their manuscript fully available?

The [PLOS Data policy](http://www.plosone.org/static/policies.action#sharing) requires authors to make all data underlying the findings described in their manuscript fully available without restriction, with rare exception (please refer to the Data Availability Statement in the manuscript PDF file). The data should be provided as part of the manuscript or its supporting information, or deposited to a public repository. For example, in addition to summary statistics, the data points behind means, medians and variance measures should be available. If there are restrictions on publicly sharing data—e.g. participant privacy or use of data from a third party—those must be specified.

Reviewer #1: Yes

Reviewer #2: No

4. Is the manuscript presented in an intelligible fashion and written in standard English?

PLOS ONE does not copyedit accepted manuscripts, so the language in submitted articles must be clear, correct, and unambiguous. Any typographical or grammatical errors should be corrected at revision, so please note any specific errors here.

Reviewer #1: Yes

Reviewer #2: Yes

**Review Comments to the Author**

Please use the space provided to explain your answers to the questions above. You may also include additional comments for the author, including concerns about dual publication, research ethics, or publication ethics. (Please upload your review as an attachment if it exceeds 20,000 characters)

**Reviewer #1:**

1. Abstract: several abbreviations used without definition.

   RK.: done
2. All manuscript: There is main focus on COPD and CF; ACO and asthma are not mentioned in an adequate manner, consider the following, with a focus on phenotypes:
   Alsayed, A. R., Abu-Samak, M. S., & Alkhatib, M. (2023). Asthma-COPD Overlap in Clinical Practice (ACO_CP 2023): Toward Precision Medicine. Journal of Personalized Medicine, 13(4), 677.
   Adrish, M., Anand, M. P., & Hanania, N. A. (2022). Phenotypes of Asthma–Chronic Obstructive Pulmonary Disease Overlap. Immunology and Allergy Clinics, 42(3), 645-655.

RK.: We appreciate this statement and added the definition of ACO (lines 152-154), referencing the two proposed papers.

3. The discussion part can be improved

   RK.: According to the criticism of both Reviewers and the Academic Editors, the discussion part was rigorously improved.

**Reviewer #2:**

I appreciated the opportunity to review " Assessment of functional diversities in patients with Asthma, Asthma-COPD overlap, and Cystic Fibrosis (CF)” by Richard Kraemer et al. Authors investigated various lung function parameters to discriminate airway disease. However, there are several concerns, mainly regarding concept of the study.

**Main concern.**

1. First, the purpose and results of the study do not match. In the discussion (Line 441 – 459), the authors mainly refer to the comparison and difference between COPD and cystic fibrosis. Cystic fibrosis is known as a risk factor for COPD, especially in young patients, so I wonder why it is necessary to evaluate the difference between cystic fibrosis and COPD rather than age-matched comparisons if authors wanted to know physiologic and functional differences between two groups. I think the main aim of the authors' research and the direction of interpretation and discussion of the results are different.

   RK.: We have difficulties to answer to this statement. By now means CF can be considered only as risk factor for COPD. This Reviewer is certainly aware of the fact, that these two diseases are completely different identities. An age-matched control is rarely possible, because the age-distributions of these 2 diseases are completely different. It was, however, thought-provoking for us, to search for the functional patterns within these two diseases.
2. Second, a definition of airway disease must be presented. In particular, ACO is only mentioned as a subtype of COPD, but ACO itself is also a disease showing heterogeneity, so it is a patient group that needs to be classified through an appropriate definition.

   RK.: We appreciate this statement and added the definition of ACO (lines 152-154), referencing the two papers proposed by Reviewer 1.
3. Table 1 presents the demographic comparison between each group. Age raises a question. Is it correct that 14.3 and 18.2 year olds are COPD and ACO patients?

   RK.: There must be a mis-understanding: The patient aged 18.2 years was considered as ACO, because she presented with both, airway obstruction (sRtot: 1.9 SDS), increased work of breathing (sWOB 2.6 SDS, sWOBex: 7.2 SDS) and a DLCO of .1.77 SDS. She was considered as potentially developing ACO. There is now patient with age 14.3 classified as ACO or COPD.

   Of course, the definition of COPD has been recently updated in GOLD, but it is necessary to specify what definition these patients were classified as COPD and ACO in their teens.
4. Regarding the implications of each lung function and plethymography parameter in airway disease, I feel that there is a lack of consideration on the meaning of indicators showing differences in asthma, ACO, COPD, and CF.

   RK.: We appreciate this statement, and focus now more on the meaning of these indicators, especially the small airways dysfunction.

**Minor comments.**

5. Introduction Line 149.
   In COPD, it is difficult to predict the prognosis only with the decline in lung function, so various prognostic risk factors for symptoms such as dyspnea, aggravation, and accompanying diseases have already been suggested. It would be better to present the analysis results on these data.

   RK.: We appreciate this statement and corrected the wording of now Line 168-173 accordingly.
6. Introduction Line 176 & Discussion line 471
   When the term ACO was first introduced in the introduction, it was presented only as an abbreviation. Conversely, the discussion explains the abbreviation of ACO. Edit is needed

   RK.: corrected
7. Line 283, Please spell out the first abbreviation. For example SDS.

   RK.: corrected
8. Discussion, Line 359-367
   The description of COPD and CF is the same as the introduction because it is very redundant.

   RK.: Thank you, this point is well taken. Wie rephrased the part accordingly

   5) Line 490-493
   It is thought that there is a limitation of generalization with a small number of retrospective studies.

   RK.: At least we found it necessary to mention this point

   6) Line 494-502
   The authors explain the research results as a physiologic biomarker that is more reliable than existing lung function parameters, but there are limitations in suggesting differences and clinical implication among diseases with appropriate cutoff values. The fact that it is a value that can be applied flexibly is felt as a disadvantage rather than an advantage.

   RK.: We appreciate

PLOS authors have the option to publish the peer review history of their article ([what does this mean?](https://journals.plos.org/plosone/s/editorial-and-peer-review-process#loc-peer-review-history)). If published, this will include your full peer review and any attached files.

RK.: yes


If you choose “no”, your identity will remain anonymous but your review may still be made public.


**Do you want your identity to be public for this peer review?** For information about this choice, including consent withdrawal, please see our [Privacy Policy](https://www.plos.org/privacy-policy).

Reviewer #1: No

Reviewer #2: No

[NOTE: If reviewer comments were submitted as an attachment file, they will be attached to this email and accessible via the submission site. Please log into your account, locate the manuscript record, and check for the action link "View Attachments". If this link does not appear, there are no attachment files.]

While revising your submission, please upload your figure files to the Preflight Analysis and Conversion Engine (PACE) digital diagnostic tool, <https://pacev2.apexcovantage.com/>. PACE helps ensure that figures meet PLOS requirements. To use PACE, you must first register as a user. Registration is free. Then, login and navigate to the UPLOAD tab, where you will find detailed instructions on how to use the tool. If you encounter any issues or have any questions when using PACE, please email PLOS at [figures@plos.org](mailto:figures@plos.org). Please note that Supporting Information files do not need this step.

RK.: All 3 Figures have been resized and uploaded to PACE

*In compliance with data protection regulations, you may request that we remove your personal registration details at any time.* [*(Remove my information/details)*](https://www.editorialmanager.com/pone/login.asp?a=r)*. Please contact the publication office if you have any questions.*

Diese Nachricht ist vertraulich. Sie darf nur von der empfangsberechtigten Person gelesen und verwendet werden. Sollten Sie diese Nachricht versehentlich erhalten haben, bitten wir Sie, den Inhalt der Nachricht vertraulich zu behandeln, uns über die Fehlleitung zu informieren und die Nachricht anschliessend zu vernichten. Jede andere Verwendung erfolgt widerrechtlich. Wir danken Ihnen für Ihr Verständnis.

This message is confidential and should only be read and used by the intended recipient. If you have received this message in error, we ask that you keep the contents confidential, inform us of the mistake and delete this message thereafter. Any other use of this message is unlawful. Thank you for your understanding.
